# Antigen-Specific Th17 Cells Are Primed by Distinct and Complementary Dendritic Cell Subsets in Oropharyngeal Candidiasis

**DOI:** 10.1371/journal.ppat.1005164

**Published:** 2015-10-02

**Authors:** Kerstin Trautwein-Weidner, André Gladiator, Florian R. Kirchner, Simone Becattini, Thomas Rülicke, Federica Sallusto, Salomé LeibundGut-Landmann

**Affiliations:** 1 Institute of Microbiology, ETH Zürich, Zürich, Switzerland; 2 Section of Immunology, Institute of Virology, University of Zürich, Zürich, Switzerland; 3 Institute for Research in Biomedicine, Università della Svizzera Italiana, Bellinzona, Switzerland; 4 Institute of Laboratory Animal Science, University of Veterinary Medicine Vienna, Vienna, Austria; Louisiana State University Health Sciences Center, UNITED STATES

## Abstract

*Candida* spp. can cause severe and chronic mucocutaneous and systemic infections in immunocompromised individuals. Protection from mucocutaneous candidiasis depends on T helper cells, in particular those secreting IL-17. The events regulating T cell activation and differentiation toward effector fates in response to fungal invasion in different tissues are poorly understood. Here we generated a *Candida*-specific TCR transgenic mouse reactive to a novel endogenous antigen that is conserved in multiple distant species of *Candida*, including the clinically highly relevant *C*. *albicans* and *C*. *glabrata*. Using TCR transgenic T cells in combination with an experimental model of oropharyngeal candidiasis (OPC) we investigated antigen presentation and Th17 priming by different subsets of dendritic cells (DCs) present in the infected oral mucosa. *Candida*-derived endogenous antigen accesses the draining lymph nodes and is directly presented by migratory DCs. Tissue-resident Flt3L-dependent DCs and CCR2-dependent monocyte-derived DCs collaborate in antigen presentation and T cell priming during OPC. In contrast, Langerhans cells, which are also present in the oral mucosa and have been shown to prime Th17 cells in the skin, are not required for induction of the *Candida*-specific T cell response upon oral challenge. This highlights the functional compartmentalization of specific DC subsets in different tissues. These data provide important new insights to our understanding of tissue-specific antifungal immunity.

## Author Summary

*Candida* spp. are present in the normal microbiota without causing damage to the host. They can become pathogenic and bear a serious health hazard for individuals with a weakened immune system. The continuous incidence of fungal infections and the increase in resistance against available antifungal drugs urge the development of novel preventive and therapeutic strategies. Knowledge gained from understanding how immunocompetent mammals control *Candida* will help develop new immunotherapeutic and-prophylactic approaches suitable to improve patient prognosis. It is well known that T helper cells, and in particular the Th17 subset, provide resistance against mucocutaneous infections with *Candida*. However, the mechanisms through which T cell-mediated antifungal immunity is induced in such context are not well understood. Here we developed a new experimental system to study the regulation of antigen-specific T cells with high resolution. Our results reveal the interplay of different dendritic cell subsets associated to the oral mucosa of infected mice that directly present fungal antigen to *Candida*-specific T cells and orchestrate a protective Th17 response in a tissue specific manner. Thus, our data highlight important features of immune regulation in the oral mucosa, a tissue that is immunologically not well characterized.

## Introduction

Opportunistic fungal infections cause an increasing medical problem due to the progression in immunosuppression worldwide [1]. *Candida* spp. present in the normal human microbiota can cause mucocutaneous infections when cellular immune barriers of the host are breached. As such, HIV^+^ individuals with low T cells counts are often affected by oropharyngeal candidiasis (OPC) [2], indicating that CD4^+^ T cells play a critical role in preventing disease symptoms. *Candida*-specific memory T helper cells are found in all healthy individuals that have been exposed to the fungus in the normal human microflora and interestingly, they belong predominantly to the subset of Interleukin 17 (IL-17)-secreting Th17 cells [3]. The notion that IL-17 plays a key role in protection from fungal infections is further supported by the identification of rare families of patients, in which inborn errors in genes linked to the IL-17 pathway are associated with chronic and recurrent forms of mucocutaneous candidiasis [4]. Although the relevance of Th17 cells in protection from *Candida* is well-documented, the regulation of these cells remains ill-defined. This gap in knowledge is entailed (among other things) by the limited information available about *Candida*-derived T cell epitopes. Out of the >10^15^ different T cell receptors (TCRs) that are theoretically generated by gene segment rearrangement, only a minute proportion recognizes *Candida*-derived antigens. The difficulty to identify these few antigen-specific T cells within the entire polyclonal repertoire hampers the study of their activation and differentiation process. The use of TCR transgenic T cells proved useful to elucidate diverse aspects of adaptive immunity in experimental systems of infectious and non-infectious diseases. However, only few TCR transgenic mouse lines specific for clinically relevant pathogens including fungi [5,6] exist to date. To circumvent this limitation, well-established TCR transgenic T cells specific for model antigens such as ovalbumin or the I-Eα chain have been used in combination with infectious agents engineered to express these model antigens. Although such systems are useful to interrogate the activation of antigen-specific T cells in the context of an infectious setting, they have important limitations, such as restricted availability, processing and presentation of model antigens during T cell priming that result in the inefficient generation of effector and memory T cells [7]. TCR transgenic T cells which recognize endogenous antigen thus represent an important advantage to functionally analyze the pathogen-specific T cell response at high resolution and in a physiological context. No TCR transgenic mouse specific for *Candida* spp. exists to date.

Differentiation of naive T cells into effector T cells depends on antigen presentation, co-stimulation and polarizing cytokines provided in *cis* by antigen presenting cells (APCs) [8]. In the context of *Candida* infection, Syk- and Card9-coupled C-type lectin receptors including Dectin-1 and Dectin-2 are relevant for the induction of Th17-inducing cytokines in response to fungal recognition [9,10]. Dectin-1 and Dectin-2 are broadly expressed by diverse subsets of mononuclear phagocytes (MNPs), many of which can potentially serve as APCs for Th17 induction. MNPs comprise monocytes, macrophages and dendritic cells (DCs). Although they are all derived from a common macrophage and DC progenitor (MDP), MNPs comprise developmentally and functionally distinct cellular subsets in different tissues, which are difficult to unambiguously distinguish on the basis of their phenotype [11]. Ly6C^hi^ monocytes differentiate from MDPs and egress from the bone marrow in a CCR2-dependent manner [12]. After entering tissues they can give rise to monocyte-derived DCs expressing high levels of CD11c and MHC II under the influence of M-CSF in inflammatory conditions [13]. MDP can also give rise to common DC progenitors (CDPs), which develop in response to Flt3 signaling and give rise to two distinct subsets of DCs: Batf3-independent and Batf3-dependent DCs, the latter of which comprises lymphoid tissue CD8α^+^ DCs and non-lymphoid-tissue CD103^+^ CD11b^-^ DCs [14]. In the skin, Langerhans cells (LCs) constitute a special case. Seeded before birth from fetal liver monocytes [15] they maintain themselves under steady state conditions by self-renewing from local precursors [16]. Non-lymphoid tissue DCs migrate from the periphery and carry antigens for presentation to T cells in the draining lymph nodes and when activated by inflammatory or infectious stimuli promote the generation of antigen-specific effector T cells.

The oral mucosa shares features with other mucosal tissues and the skin, but it constitutes a unique tissue with its own cellular composition and function [17]. LCs, CD103^+^ CD11b^-^ DCs and CD11b^+^ CD103^-^ DCs have all been identified, but their role in immune activation is not well understood. In addition, inflammatory monocytes that give rise to monocyte-derived DCs infiltrate the oral mucosa upon infection and inflammation. Using an experimental model of OPC we show here how these different DC subsets orchestrate the T cell response during oral infection. We made use of a novel TCR-transgenic mouse, whose T cells specifically recognize an endogenous *Candida*-derived antigen, to functionally determine the presentation capacity of individual APC subsets. We found that both monocyte-derived and Flt3L-dependent conventional DCs carry fungal antigen from the site of infection to the draining cervical lymph nodes where they directly present it to T cells. In a partially redundant manner they instruct the activation and differentiation of *Candida*-specific T cells into cytokine-producing effector cells *in vivo*. This indicates that the initiation of an antifungal Th17 response depends on an intricate interplay of different APC subsets in the oral mucosa allowing the generation of a robust response.

## Materials and Methods

### Ethics statement

All mouse experiments described in this study were conducted in strict accordance with the guidelines of the Swiss and Austrian Animal Protection Law and were performed under protocols approved by the Veterinary office of the Canton Zürich, Switzerland (license number 184/2009 and 201/2012) and by the institutional ethics and animal welfare committee of the University of Veterinary Medicine Vienna (license number 68.205/0258-II/3b/2011). All efforts were made to minimize suffering and ensure the highest ethical and humane standards.

### Mice and depletion strategies

C57BL/6J mice (B6) were purchased from Janvier Elevage. *Ccr7*-/- [18], *Batf3*-/- [19], *Ccr2*-/- [20] and *Flt3l*-/- [21] were bred at the Laboratory Animal Service Center (University of Zürich, Switzerland). Langerin-DTR mice [22] were a kind gift from Björn Claussen and Dr. Kordula Kautz-Neu (Mainz, Germany). All mice were on the C57BL/6 background, kept in specific pathogen-free conditions and used at 6–15 weeks of age. In some experiments, mice were treated with diphtheria toxin via the intraperitoneal route (10 ng per gram body weight, daily starting from 1 day prior to infection to day +2 post-infection). For blocking of CSF1R, 2 mg anti-CSF1R antibody (clone AFS98, kindly provided by Melanie Greter, Zürich) was administered intraperitoneally one day prior infection, followed by a second dose of 1 mg on day 1 post-infection.

### Generation of the TCR transgenic mouse line Hector

Splenocytes were isolated from systemically infected B6 mice (infection on day -24 and day -10) and re-activated with GM-CSF-induced bone marrow-derived DCs at a ratio 10:1 in the presence of 2 x 10^4^ heat-killed *C*. *albicans* yeast cells. After 3 days cells were fused with BW5147 lymphoma cells (ATCC #TIB48) using polyethylene glycol 1500 (AppliChem) [23] selected in hypoxanthine, aminopterin and thymidine (HAT) medium (Invitrogen). Specificity of the CD4^+^ T cell hybridoma for *C*. *albicans* was assessed by co-culturing 5 x 10^4^ hybridoma cells with 5 x 10^4^ DC^1940^ cells that were pulsed with 5 x 10^4^ heat-killed fungi. After 24h supernatants were transferred to 1x10^4^ CTLL-2 and their viability was assessed by the alamar blue cell viability test (Invitrogen) following the manufacturer’s instructions. *C*. *albicans*-specific hybridoma were subcloned by serial dilution to generate the monoclonal hybridoma cell line 59.8, which was re-screened for specificity. TCR Vα2 and Vβ4 expression was determined by flow cytometry. RNA was isolated form the hybridoma using TRI reagent (Sigma) according to the manufacturer's instructions, and cDNA was generated using M-MLV Reverse Transcriptase RNase, H- (Promega). cDNA was amplified with a TCRα-specific primer set [24] and a TCRβ-specific primer set [25]. Sequencing of the PCR products was done by Microsynth and then aligned to the mouse genome using Ensemble database (http://www.ensembl.org/Mus_musculus) and analyzed with Immunogenetics Information System (www.imgt.org). The identified Vα2Jα53 and Vβ4Dβ1Jβ1 gene segments were amplified from the genomic DNA using the following primers: Vα2 fwd, 5'-tgacccgggagcttcagtctaggaggaatg-3'; Vα2 rev, 5'-atatcggccgctcctgtaatacttacttg-3'; Vβ4 fwd, 5'-tgtctcgagagagatcctatcctgtgtgacactgctatg-3'; Vβ4 rev, 5'-tgcccgcggcatcccacacccaaagaccctcaggccttaccta-3'; digested with *XmaI* and *NotI* or *XhoI* and *SacII*, respectively, and cloned into previously described TCR expression vectors [26]. The resulting pTαVα2 and pTβVβ4 were digested with *SalI* respectively *KpnI* to excise the transgenes from prokaryotic vector DNA. The isolated linearized fragments were co-injected in equimolar ratios into fertilized C57BL/6N oocytes according to the standard method [27]. The resulting TCR transgenic mouse line selected for experimental use was designated according to the standardized genetic nomenclature for mice: C57BL/6N-Tg(TcraTcrb)603Biat (Hector) [28]. It was backcrossed to express the congenic marker Thy1.1 and bred at our animal facility Rodent Center HCI.

### Fungal strain and infections

The *C*. *albicans* laboratory strain SC5314 was used throughout unless stated otherwise. Clinical isolates of *C*. *albicans*, *C*. *dubliniensis*, *C*. *krusei*, *and C*. *glabrata* were obtained from Cristina Fragoso and Orlando Petrini (Bellinzona, Switzerland). All fungal strains were grown in YPD medium at 30°C for 15–18 hours. Mice were infected with 2.5 x 10^6^ cfu *C*. *albicans* sublingually as described [29] without immunosuppression. In some experiments, mice were treated with 400 μg Fluconazole (Ratiopharm) intraperitoneally on day 2 post-infection and 0.2 mg/ml Fluconazole (Sigma-Adrich) in the drinking water from day 2 post-infection until the mice were sacrificed to prevent fungal overgrowth, which may affect the degree of the T cell response. Mice were monitored for morbidity and weight throughout the course of all experiments. Determining the body weight of infected mice represents a sensitive method for monitoring the control of infection. While all mice sublingually infected with *C*. *albicans* strain SC5314 lose 10–15% of their body weight within the first 2 days post-infection, their recovery of the original weight within 5–7 days post-infection correlates with rapid fungal elimination [30]. For determination of fungal burden, the tongue of euthanized animals was removed, homogenized in sterile 0.05% NP40 in H_2_O for 3 minutes at 25 Hz using a Tissue Lyzer (Qiagen) and serial dilutions were plated on YPD agar containing 100 μg/ml Ampicillin. For systemic infection, mice were injected intravenously with 5 x 10^4^ cfu *C*. *albicans*.

### Immunizations

Mice were immunized subcutaneously with 50 μg pADH1_126-140_ (EMC microcollection) in Incomplete Freund's Adjuvant (IFA, Sigma) mixed with 25 μg CpG (Microsynth).

### Preparation of mannoprotein extract

*C*. *albicans* strain ATCC14053 was grown YPD medium at 30°C for 16 hours, washed extensively and resuspended in 20mM Na citrate buffer. Samples were autoclaved for 1.5h at 121°C and spun at max speed for 15’. The supernatant, containing highly soluble mannoproteins, was harvested and stored at -20°C. A mix of equal volumes of Fehling solution I (7% hydrate copper(II)sulfate in 100ml H2O) and Fehling solution II (35% potassium tartrate + 10% NaOH in 100 ml H2O) was prepared and added to the thawed supernatant in a 1:1 ratio for 30’. After centrifugation for 15’ at max speed a pellet was obtained that derived from precipitation of mannoproteins. The pellet was dissolved in 3N HCl. Proteins were precipitated upon addition of 8:1 MetOH + Acetic acid, incubation for 1h on a rotating wheal at 4°C and centrifugation (step repeated twice). Finally, two steps of wash/dehydration were performed with MetOH and Ether, respectively. Pellets were dried with a vacuum pump and stored at -80C. Samples were resuspended in water and quantified using Bradford reagent (Biorad) prior to use.

### Cell lines

The *C*. *albicans*-specific T cell hybridoma 59.8 was maintained in DMEM medium supplemented with 10% FCS, Penicillin/Streptomycin, 2mM Glutamine and 50 μM 2-Mercaptoethanol. The DC cell line DC^1940^ [31] was kept in IMDM medium, supplemented with 10% FCS, Penicillin/Streptomycin, 2 mM Glutamine and 50 μM 2-Mercaptoethanol. CTLL-2 cells, which are dependent on IL-2 for growth [32], were maintained in RPMI 1640 medium supplemented with 10% FCS, Penicillin/Streptomycin and 2mM Glutamine and recombinant IL-2.

### Isolation of tongue cells

Mice were anaesthetized with a sublethal dose of Ketamine (100 mg/kg), Xylazin (20 mg/kg) and Acepromazin (2.9 mg/kg), and perfused by injection of PBS into the right heart ventricle. Tongues were removed, cut into fine pieces and digested with DNase I (2.4 mg/ml, Roche) and Collagenase IV (4.8 mg/ml, Invitrogen) in PBS for 45 min at 37°C. Single cell suspensions were enriched for leukocytes using 30% Percoll and analyzed by flow cytometry.

### Isolation of APC populations from cervical lymph nodes

Cervical lymph nodes were removed from infected mice on day 2 post-infection or from naïve controls and digested with DNase I (2.4 mg/ml, Roche) and Collagenase I (2.4 mg/ml, Invitrogen) in PBS for 15 min at 37°C. CD11b^+^ cells were enriched with the help of biotinylated anti-CD11b antibody and Streptavidin microbeads (Miltenyi) according to the manufacturer's recommendations.

### Hybridoma activation assay

5 x 10^4^ *C*. *albicans*-specific T cell hybridoma 59.8 was co-cultured with 5 x 10^4^ DC^1940^ cells that were pulsed with 5 x 10^4^ heat-killed *C*. *albicans*, mannoprotein extract, 1 μg/ml of a pool of overlapping 15-mer peptides covering the entire ADH1 protein sequence or with 1 μg/ml of individual peptides (A&A, La Jolla, CA). Alternatively, 10^5^ 59.8 hybridoma cells were stimulated with 10^5^ cervical lymph node cells from sublingually infected mice, whichwere enriched for CD11b^+^ cells, without addition of exogenous antigen. After 24h of co-culture at 37°C, IL-2 production by the hybridoma cells was quantified with the CTLL-2 bioassay. For this, 1 x 10^4^ CTLL-2 cells were incubated with supernatant from the hybridoma overnight and their viability was assessed by the alamar blue cell viability test (Invitrogen) following the manufacturer’s instructions. As a control, CTLL-2 cells were incubated with recombinant IL-2 or with medium alone.

### *In vitro* activation and proliferation of Hector T cells

CD4^+^ T cells were purified from spleen (and in some cases from spleen and lymph nodes) of TCR transgenic Hector mice with anti-CD4 microbeads (Miltenyi) following the manufacturer's recommendations. In some cases, they were labeled with 1 μM carboxyfluorescein succinimidyl ester (CFSE, Invitrogen) for 5 minutes at room temperature. 6 x 10^4^ T cells were then co-cultured with 1 x 10^4^ CD11b^+^-enriched or FACS-sorted cervical lymph node cells from sublingually infected mice without addition of exogenous antigen. Alternatively, 6 x 10^4^ Hector T cells were co-cultured with 1 x 10^4^ splenocytes that had been pulsed with 100ng/ml of a pool of overlapping 15-mer peptides covering the entire ADH1 protein sequence or with 100ng/ml pADH1_126-140_. Expression of CD69 as a marker of T cell activation was analyzed by flow cytometry after 24 hours of co-culture. T cell proliferation was determined by measuring dilution of the CFSE signal by flow cytometry after 3 to 4 days.

### Analysis of Th17 priming *in vivo*

In some experiments, 1 x 10^6^ CD4^+^ Hector T cells were prepared as described above and adoptively transferred into recipients one day prior to infection. On day 7 post-infection, cervical lymph nodes were removed and single cell suspensions were prepared by digested with DNase I (2.4 mg/ml, Roche) and Collagenase I (2.4 mg/ml, Invitrogen) in PBS for 15 min at 37°C. For inducing cytokine secretion by primed T cells, 10^6^ cervical lymph node cells were then re-stimulated for 6 hours with 1 x 10^5^ DC^1940^ cells pulsed with pADH1_126-140_ peptide (100 ng/ml), 2.5x10^5^/ml heat-killed *C*. *albicans* or left unpulsed. Brefeldin A (10 μg/ml, AppliChem) was added for the last 5 hours. IL-17 production by endogenous CD3^+^ CD4^+^ T cells and/or CD3^+^ CD4^+^ Thy1.1^+^ Vα2^+^ Hector T cells was then analyzed by flow cytometry.

### Flow cytometry

All antibodies were from Biolegend, if not stated otherwise. For flow cytometry analysis of APCs, single cell suspensions of cervical lymph nodes and tongue were prepared as described before and stained in PBS with LIVE/DEAD Fixable Near-IR Stain (Life Technologies), CD45.2 (clone 104,), CD11b (clone M1/70), Ly6C (clone AL-21, BD Biosciences), Ly6G (clone 1A8), I-A/I-E (clone M5/114.15.2), CD11c (clone N418), CCR2 (clone 475301, R&D Biosystems), Langerin (clone eBioL31, eBiosciences), CD103 (clone 2E7), CD64 (clone X54-5/7.), CD24 (clone M1/69) or SIRPα (clone P84). For flow cytometry analysis of T cells, single cell suspensions were stained in ice-cold PBS with LIVE/DEAD Fixable Near-IR Stain, CD4 (clone RM4-5), CD3ε (clone 145-2C11), and in some cases CD44 (clone IM7), CD62L (clone MEL-14) and/or CD69 (clone H1.2F3). Thy1.1 (clone OX-7) and TCRVα2 (clone B20.1) were added for identification of Hector TCR transgenic T cells. For intracellular cytokine staining, T cells were first incubated in ice-cold PBS containing LIVE/DEAD Fixable Near-IR Stain and surface marker antibodies. After fixation and permeabilization using BD Cytofix/Cytoperm (BD Biosciences) the cells were then incubated in in Perm/Wash buffer (BD Biosciences) containing anti-IL-17A (clone TC11-18H10.1) and anti-IFNγ (clone XMG1.2) antibodies. Data were acquired on a LSRII (BD Biosciences) and analyzed with FlowJo software (Tristar). For all experiments, the data were pre-gated on live single cells.

For isolating APC subsets by FACS sorting, cervical lymph nodes single cell suspensions were depleted of B and T cells with the help of biotinylated anti-CD19 (clone 6D5) and anti-CD3ε and Streptavidin microbeads (Miltenyi) according to the manufacturer's recommendations, stained in ice-cold PBS with LIVE/DEAD Fixable Near-IR Stain, Ly6G, I-A/I-E, CD11c and CCR2, and sorted on a FACSAriaII (BD Biosciences).

### Statistics

Statistical significance was determined by student's t-test using GraphPad Prism (GraphPad Software) with *, p < 0.05; **, p < 0.01; ***, p < 0.001. For data plotted on a logarithmic scale the geometric mean is indicated.

## Results

### Generation of a TCR transgenic mouse reactive to a novel *Candida*-derived T cell epitope

To study the immune response to *Candida* antigen, we first developed a *Candida*-specific TCR transgenic mouse. In this mouse, dubbed 'Hector', 40–60% of all peripheral CD4^+^ T cells expressed a transgenic TCR consisting of Vα2 and Vβ4 genes sequenced from the T cell hybridoma 59.8, which was generated from T cells that were isolated from a *C*. *albicans-*infected C57BL/6J (B6) mouse (S1 Fig).

**Fig 1:**
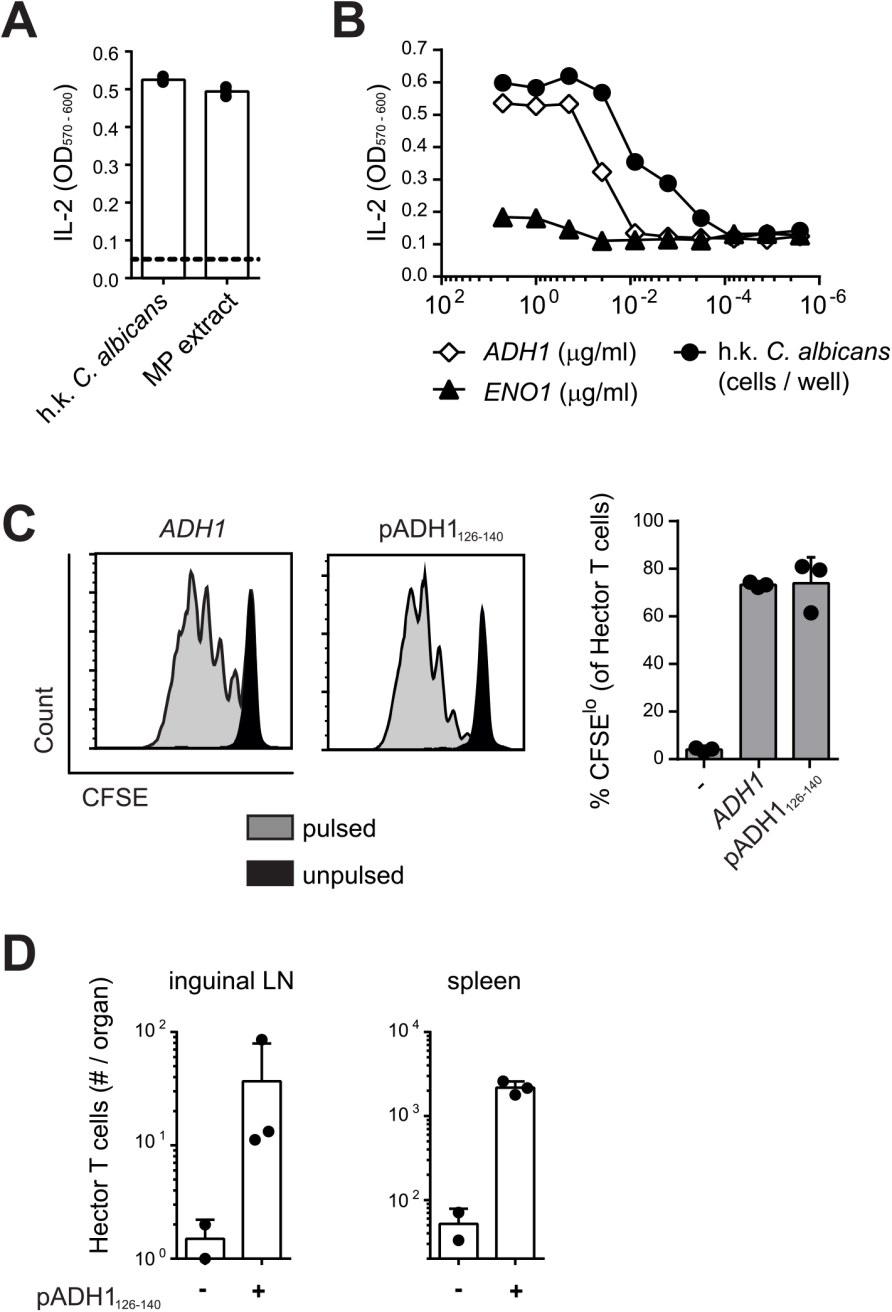
Hector T cells recognize residues 126–140 of *C*. *albicans* ADH1. (**A—B**) Hybridoma cells were stimulated with DC^1940^ cells that were pulsed with heat-killed (h.k.) *C*. *albicans*, mannoprotein (MP) extract (A) or a pool of overlapping 15-mer peptides covering the entire ADH1 and ENO1 protein sequences (B). IL-2 secretion was quantified with the CTLL2 bioassay. (**C**) CFSE-labelled CD4^+^ Hector T cells were stimulated with splenocytes that were pulsed with a pool of overlapping 15-mer peptides covering the entire ADH1 protein sequence or with peptide C2 (pADH1_126-140_), or that were left unpulsed. Proliferation was analyzed by flow cytometry after 4 days of co-culture. Representative FACS plots are shown on the left, a summary graph with pooled data is shown on the right. (**D**) CD4^+^ Hector T cells were adoptively transferred into B6 mice one day before they were immunized subcutaneously in the flank with IFA and 25μg CpG mixed with or without 50μg peptide C2 (pADH1_126-140_) as indicated. The expansion of Hector T cells in the inguinal lymph nodes (LN, left) and in the spleen (right) was analyzed by flow cytometry on day 7 post-immunization. Each symbol represents one mouse, the mean + SD is indicated. All data are representative of at least 2 independent experiments.

The antigenic specificity of Hector T cells was determined using the T cell hybridoma 59.8 and different *C*. *albicans* antigenic preparations presented by the DC^1940^ cell line [31]. As positive control, the T cell hybridoma 59.8 exposed to *C*. *albicans*-loaded DC^1940^ cells produced IL-2, which was quantified using the CTLL-2 bioassay (Fig 1A). The hybridoma was found to react against DC^1940^ cells pulsed with a mannoprotein-enriched fraction (Fig 1A), indicating that the antigenic determinant was present in the *C*. *albicans* cell wall. Mass spectrum analysis of the mannoprotein extract revealed the presence of five abundant proteins (yeast wall protein1, YWP1; enolase, ENO1; glyceraldehyde-3-phosphate dehydrogenase, G3PDH; alcohol dehydrogenase, ADH1; fructose bisphosphate 1, FBA1). A peptide pool, consisting of 15-mers overlapping by 10 amino acids covering the entire ADH1 sequence, stimulated IL-2 production from the hybridoma 59.8 in a dose-dependent manner (Fig 1B), while no response was detected against peptide pools covering the sequences of YWP1, ENO1, G3PDH or FBA1. By screening the individual peptides of ADH1, we identified 3 peptides that triggered IL-2 production by the hybridoma (S2 Fig). Of these, peptide C2 induced the strongest when tested for their capacity to induce proliferation of Hector T cells, while peptide C3 induced a much weaker response and peptide D1 failed to induce a response in this assay (S2 Fig). Peptide C2 stimulated proliferation of Hector T cells to an extent that was comparable to that induced by the peptide pool covering the entire ADH1 sequence (Fig 1C). Finally, the specificity of Hector T cells for the ADH1 peptide C2 was confirmed *in vivo* in mice adoptively transferred with CD4^+^ Hector T cells and immunized with the peptide admixed with CpG adjuvant (Fig 1D).

Peptide C2 mapped to residues 126–140 of ADH1 (pADH1_126-140_), corresponding to sequence GSFEQYATADAVQAA, and was predicted to have a good binding affinity to I-A^b^ (IC_50_ = 605, SMM align method) [33]. In line with the high degree of conservation of the ADH1 protein and in particular of the pADH1_126-140_ peptide sequence across *Candida* spp., we found a comparable dose-dependent response of hybridoma 59.8 to different strains of *C*. *albicans*, *C*. *dubliniensis*, *C*. *tropicalis*, *C*. *glabrata* and *C*. *krusei* (S2 Fig). Moreover, the epitope was also conserved in *S*. *cerevisiae* (S2 Fig), but not in other ascomycetes such as Aspergillus or the dimorphic fungi. Thus, the Hector mouse is a source of T cells highly enriched in CD4^+^ T cells specific for a novel ADH1-derived antigen and they provide a valuable tool for studying in detail and in an antigen-specific manner the adaptive immune response to *C*. *albicans*.

### Oral *C*. *albicans* infection induces differentiation to Th17 of endogenous and TCR transgenic *C*. *albicans*-specific CD4 T cells

**Fig 2:**
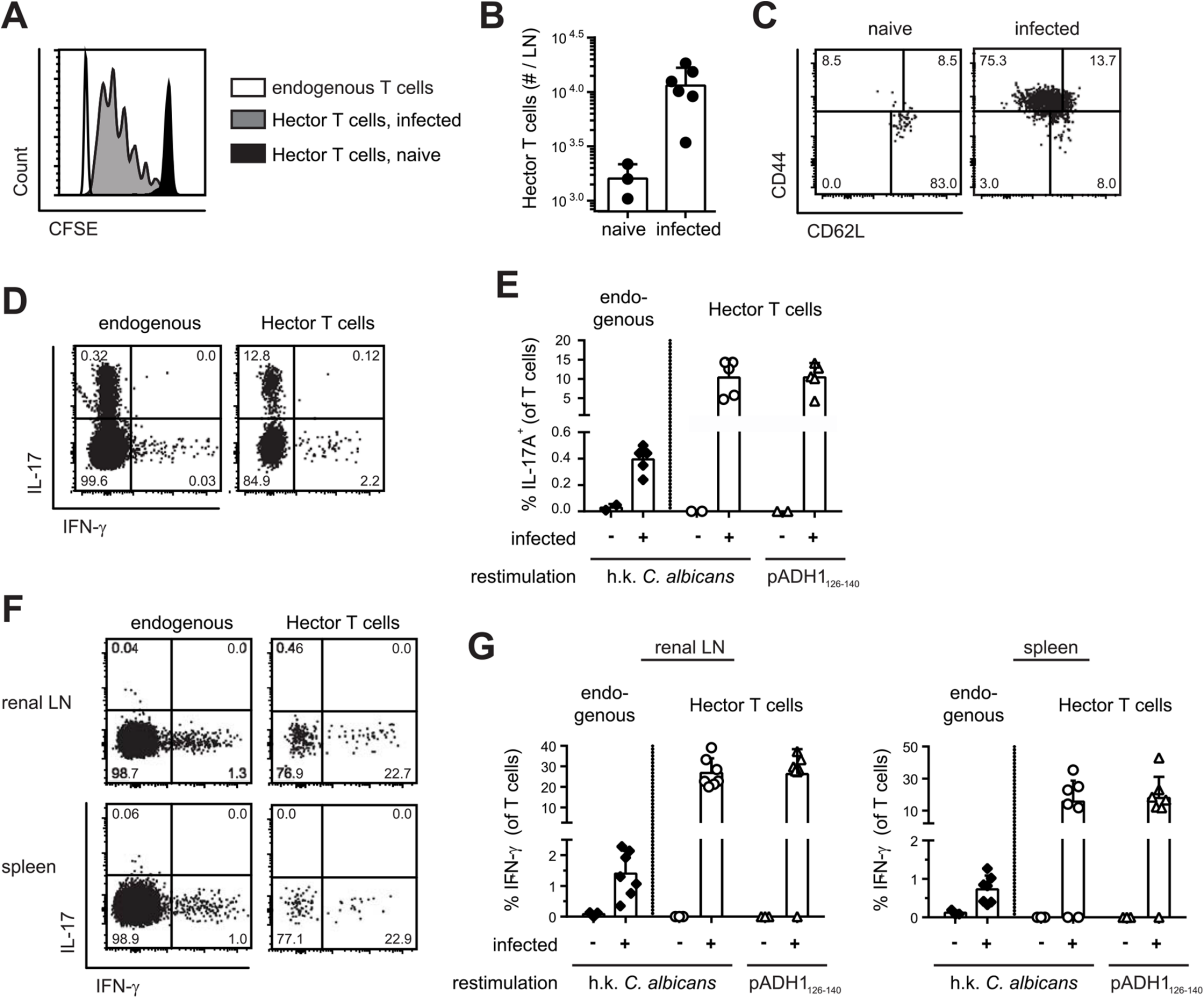
Antigen-specific Th cell response to *C*. *albicans* oropharyngeal infection. (**A, B**) CFSE-labelled CD4^+^ Hector T cells were adoptively transferred one day prior to sublingual infection of B6 mice with *C*. *albicans*. Proliferation of Thy1.1^+^ CD4^+^ Vα2^+^ Hector cells was analyzed by flow cytometry on day 3 post-infection. A representative FACS plot is shown in (A), quantification of Thy1.1^+^ CD4^+^ Vα2^+^ Hector cells in cervical lymph nodes is shown in (B). Each symbol represents an individual mouse, the mean + SD is indicated, data are pooled form 2 independent experiments. (**C—E**) CD4^+^ Hector T cells were adoptively transferred one day prior to sublingual infection with *C*. *albicans*. In (C), Thy1.1^+^ CD4^+^ Vα2^+^ Hector cells in the cervical lymph nodes were analyzed on day 7 post-infection for expression of CD44 and CD62L. Representative FACS plots from 3 independent experiments are shown. In (D—E), cytokine production by endogenous CD3^+^ CD4^+^ Thy1.2^+^ T cells (left) and Thy1.1^+^ CD4^+^ Vα2^+^ Hector cells (right) on day 7 post-infection was analyzed by flow cytometry after re-stimulation with DC^1940^ cells pulsed with heat-killed (h.k.) *C*. *albicans* or pADH1_126-140_ as indicated. Representative FACS plots are shown in C and D, quantification of IL-17-producing cells is shown in (E). Each symbol represents an individual mouse, the mean + SD is indicated, data are pooled form 2 independent experiments. (**F, G**) B6 mice obtained an adoptive transfer of CD4^+^ Hector T cells one day prior to systemic infection with 5 x 10^4^ cfu *C*. *albicans*. Cytokine production by endogenous CD3^+^ CD4^+^ Thy1.2^+^ T cells (left) and Thy1.1^+^ CD4^+^ Vα2^+^ Hector cells (right) in the renal lymph nodes (left) and spleen (right) on day 7 post-infection was analyzed after re-stimulation with DC^1940^ cells pulsed with heat-killed (h.k.) *C*. *albicans* or pADH1_126-140_ as indicated. Representative FACS plots are shown in (F), quantification of IFN-γ-producing cells is shown in (G). Each symbol represents an individual mouse, the mean + SD is indicated, data are pooled form 2 independent experiments.

With the availability of *C*. *albicans*-specific TCR transgenic T cells, we set out to study the immune response to *C*. *albicans* antigen in a model of oral mucosal infection. CFSE-labeled CD4^+^ Hector T cells were adoptively transferred into B6 mice that were then infected sublingually with 2.5 x 10^6^ cfu *C*. *albicans*, as previously described [29]. As shown in Fig 2, Hector T cells proliferated extensively in the draining cervical lymph nodes upon infection (Fig 2A and 2B), displayed an activated CD44^hi^ CD62^lo^ phenotype (Fig 2C), and differentiated to Th17 cells (Fig 2D and 2E). Cytokine production by Hector T cells was comparable whether the cells were re-stimulated with heat-killed fungus or with the cognate antigen (Fig 2D and 2E). Moreover, similar results were obtained irrespective of whether 10^5^ or the standard dose of 10^6^ Hector T cells were adoptively transferred (S3 Fig). A sizable proportion of endogenous polyclonal T cells (0.2 to 0.4% of the CD3^+^CD4^+^ cells) also differentiated to Th17 cells, most of which co-produced IL-17A, IL-17F and IL-22, while IFN-γ-producing cells could barely be detected. (Fig 2D and 2E). In contrast to the oral infection, systemic infection with *C*. *albicans* induced differentiation of both Hector T cells and endogenous T cells into IFN-γ-producing Th1 cells (Fig 2F and 2G).

### Transport of *C*. *albicans*-derived antigen to the cervical lymph nodes is mostly CCR7-dependent

**Fig 3:**
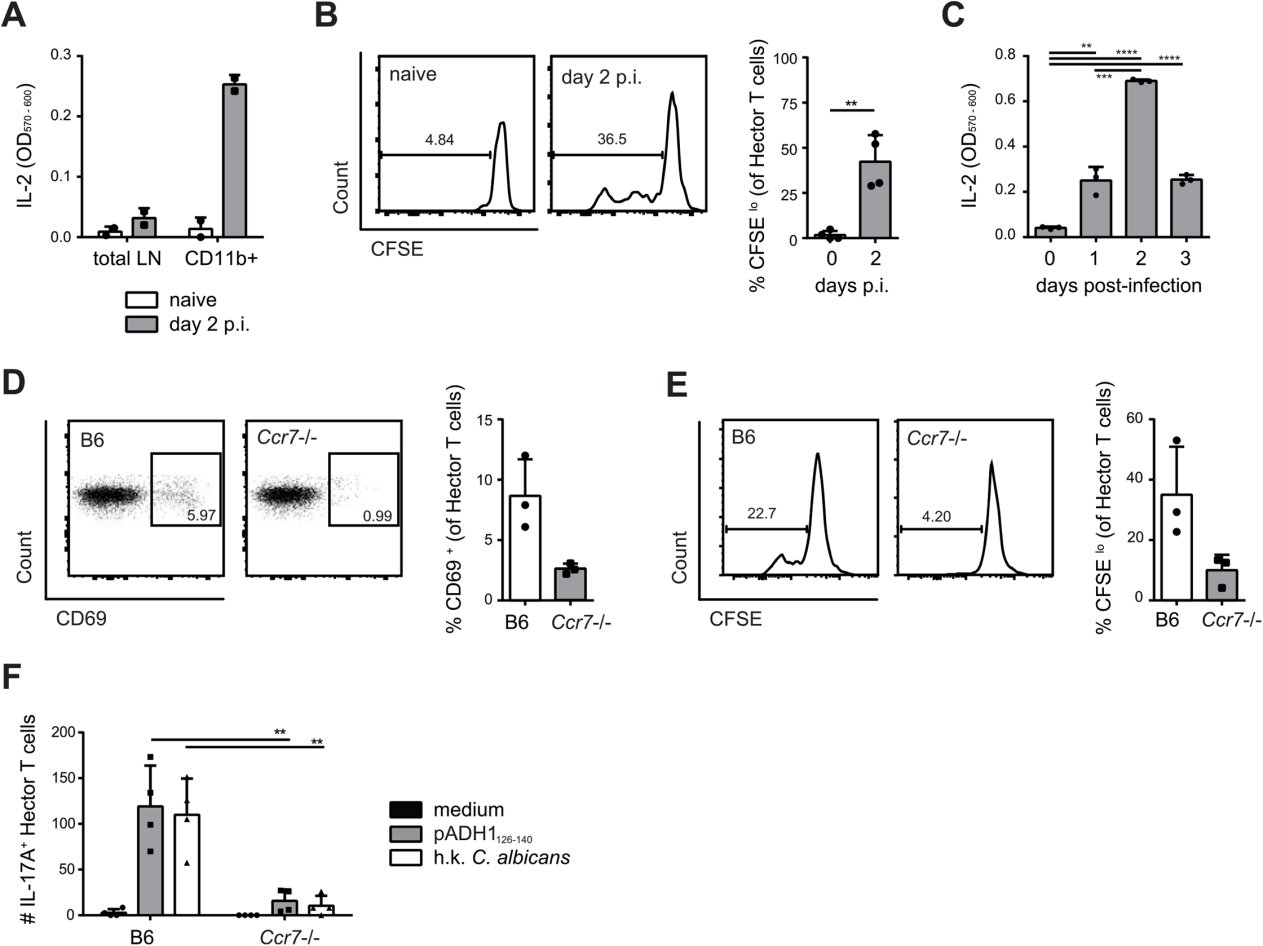
Transport of *C*. *albicans*-derived antigen to the cervical lymph nodes is CCR7-dependent. (**A**) Cervical lymph node cells that were isolated from naïve or from sublingually infected mice on day 2 post-infection and either enriched for CD11b^+^ cells or left non-enriched were co-cultured with the *C*. *albicans*-specific T cell hybridoma cells. IL-2 production as a read-out for hybridoma activation was quantified by CTLL-2 bioassay. (**B**) Cervical lymph node cells were isolated from naïve mice or from sublingually infected mice on day 2 p.i., enriched for CD11b^+^ cells, and co-cultured with CFSE-labeled CD4^+^ Hector T cells. CFSE-dilution of the Thy1.1^+^ CD3^+^ CD4^+^ Hector cells was analyzed after 4 days. (**C**) CD11b^+^ cells were enriched from cervical lymph nodes at different time points after infection as indicated and analyzed for antigen presentation as described in (A). **(D—E)** cervical lymph node cells were isolated from sublingually infected B6 or *Ccr7*-/- mice on day 2 post-infection, enriched for CD11b^+^ cells and co-cultured with CD4^+^ Hector T cells. CD69 expression (D) and CFSE dilution (E) of Thy1.1^+^ CD3^+^ CD4^+^ TCRVα2^+^ cells was analyzed on day 1 and day4 respectively. In (B), (D) and (E), representative FACS plots are shown on the left; summary of data from individual mice with mean + SD are shown on the right. Data are from individual experiments that are representative of at least two independent experiments each. **(F)** CD4^+^ Hector T cells were adoptively transferred into B6 and *Ccr7*-/- mice one day prior to sublingual infection. Cytokine production by Thy1.1^+^ CD3^+^ CD4^+^ Hector cells in the cervical lymph nodes was analyzed on day 7 post-infection after re-stimulation with DC^1940^ cells pulsed with pADH1_126-140_, heat-killed (h.k.) *C*. *albicans* or left unpulsed as indicated. Symbols represent individual mice pooled from 2 independent experiments, the mean + SD is indicated.

We next asked how in OPC *C*. *albicans*-derived antigen can reach the draining lymph node from the site of infection, since the fungus is normally restricted to the keratinized layer of the oral epithelium and does not invade deeper tissues or drain to lymphoid organs in immunocompetent mice [30]. Indeed, we were unable to culture *C*. *albicans* from the cervical lymph nodes of infected B6 mice. A likely possibility is that *C*. *albicans*-derived antigen accesses draining lymph nodes transported by migratory cells that arrive from the site of infection to the lymph nodes through afferent lymphatics. To test this possibility, we first enriched CD11b^+^ cells from cervical lymph nodes of *C*. *albicans* infected and naïve mice and found that only CD11b^+^ cells from infected mice could directly and rapidly stimulate the T cell hybridoma 59.8 (Fig 3A) and induce activation and proliferation of Hector T cells *in vitro* (Fig 3B). Kinetic studies showed that maximal presentation was achieved on day 2 post-infection (Fig 3C). We then enriched CD11b^+^ cells from cervical lymph nodes of infected B6 and *Ccr7* ^–/–^mice, in which migration of cells from the periphery to draining lymph nodes is severely impaired [34]. Strikingly, activation of Hector T cells was strongly reduced (Fig 3D and 3E).

To further corroborate these data, we adoptively transferred CCR7-sufficient Hector T cells into *Ccr7* ^–/–^or B6 mice prior to sublingual infection with *C*. *albicans*. Expression of CCR7 on Hector T cells allows their normal entry into cervical lymph nodes via high endothelial venules. When lymph nodes were analyzed on day 7 post-infection, we found that Hector T cells differentiated into IL-17A-secreting effector cells in response to *C*. *albicans* in B6 mice, but their expansion and differentiation was strongly reduced in CCR7-deficient mice (Fig 3F), indicating that the delivery of *C*. *albicans*-derived antigen to the cervical lymph nodes was cell-associated and dependent on CCR7-mediated cell trafficking *in vivo*. Attempts to visualize the cells that take up *C*. *albicans* (either labeled with a fluorescent dye or engineered to express GFP) failed both in the tongue or in the cervical lymph nodes of infected mice, presumably due to the fact that the majority of *C*. *albicans* hyphae, the predominant morphotype in infected oral tissue, remained extracellular and the ingested material was degraded rapidly by the phagocytosing cells.

### MHC II^hi^ CD11c^+^ migratory DCs present *C*. *albicans*-derived antigen in the cervical lymph nodes

**Fig 4:**
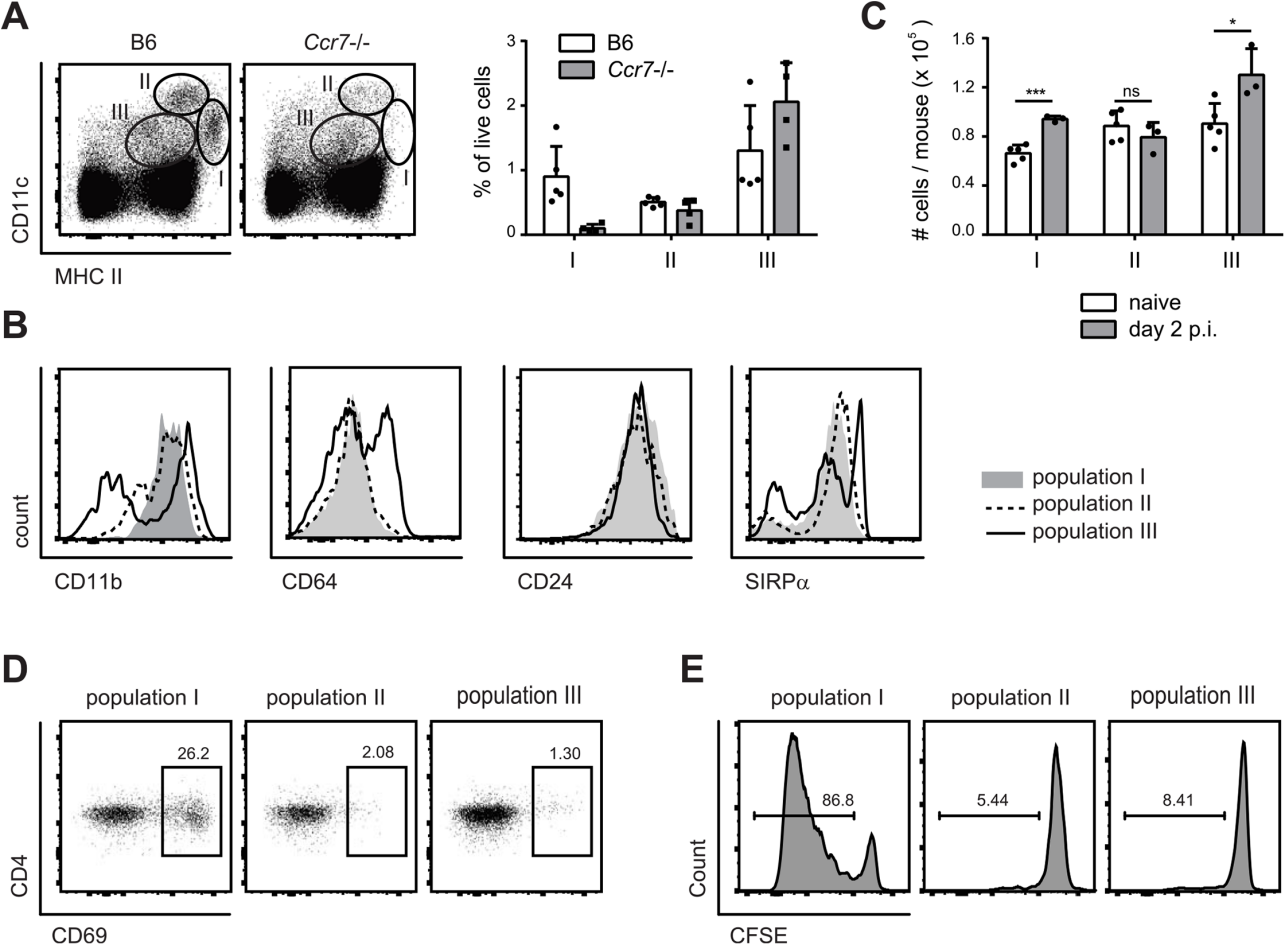
MHC II^hi^ CD11c^+^ migratory DCs present *C*. *albicans*-derived antigen in the cervical lymph nodes. (**A**) Three major populations of CD11c and MHC II positive cells were identified in the cervical lymph nodes of B6 and *Ccr7*-/- mice on day 2 post-infection: population I (MHC II^high^ CD11c^+^), population II (MHC II^+^ CD11c^high^) and population III (MHC II^+^ CD11c^+^). Representative FACS plots are shown on the left, summary of data from individual mice with mean + SD are shown on the right. (**B**) CD11b, CD64, CD24 and SIRPα expression in each of the three DC populations in cervical lymph nodes on day 2 post-infection, as defined in (A). Representative FACS plots are shown. **(C)** The three DC populations defined in (A) were quantified in the cervical lymph nodes of naïve and OPC infected B6 mice. Each symbol represents an individual mouse. Data in (A—C) are representative of at least two independent experiments. (**D—E**) Mice were infected sublingually with *C*. *albicans* and cervical lymph nodes were isolated on day 2 post-infection. The three DC populations defined in (A) were FACS-sorted and co-cultured with CD4^+^ Hector T cells without adding additional exogenous antigen. Thy1.1^+^ CD3^+^ CD4^+^ TCRVα2^+^ cells were analyzed for CD69 expression after 1 day (D) and for dilution of the CFSE signal after 4 days (E). Data show representative FACS plots from at least 3 independent experiments.

Three major populations of DCs could be identified in cervical lymph nodes according to the expression of MHC II and CD11c: Population I (MHC II^high^ CD11c^+^), population II (CD11c^high^ MCH II^+^), and population III (CD11c^int^ MHC II^int^) (Fig 4A). Population I and II expressed CD11b, CD24 and SIRP1α but not CD64, while population III was more heterogeneous for expression of some of these markers (Fig 4B). When compared to the populations found in lymph nodes of *Ccr7* ^*–/–*^mice, population II could be identified as lymph node resident DCs and population I as migratory DCs. Population III could also be identified as composed mainly of lymph node resident cells, since the number of CD11c^int^ MHC II^int^ cells was not affected in *Ccr7* ^*–/–*^mice (Fig 4A). During the course of *C*. *albicans* infection, populations I and III increased, while population II remained unchanged (Fig 4C).

To define the antigen-presenting capacity in OPC, we FACS-sorted to high purity the three DC populations from cervical lymph nodes of *C*. *albicans* infected mice and directly tested their capacity to activate Hector T cells *in vitro* without addition of exogenous antigen. We also tested Ly6G^+^ neutrophils isolated from the cervical lymph nodes of the same mice in the assay, since previous studies have shown that these cells can present antigen in certain conditions [35]. Hector T cells responded only very weekly, or not at all, to CD11c^hi^ MHC II^int^ DCs (population II), CD11c^int^ MHC II^int^ cells (population III) and Ly6G^+^ neutrophils. In contrast, they rapidly upregulated CD69 and proliferated strongly when co-cultured with MHC II^hi^ CD11c^+^ migratory DCs (population I) (Fig 4D and 4E). In the assay, the T cell stimulatory capacity was strictly dependent on *C*. *albicans*-derived antigen, since DCs isolated from naïve animals were unable to induce proliferation of Hector T cells. Together with the previous findings, these data indicate that cells within the MHC II^hi^ CD11c^+^ migratory population can capture *C*. *albicans*-derived antigen from the peripheral tissue and transport them to the cervical lymph nodes where they are presented to CD4^+^ T cells. Consistent with a role of migratory DCs in the induction of Th17 differentiation, cells within population I produced higher levels of IL-6 compared to the other two DC populations in the cervical lymph nodes of OPC-infected mice (S4 Fig).

### Flt3L-dependent migratory DCs and monocyte-derived DCs present *C*. *albicans*-derived antigen

**Fig 5:**
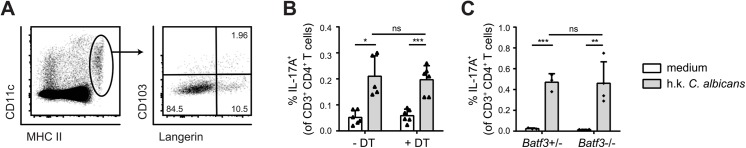
Langerin^+^ cells are not essential for efficient Th17 priming. **(A)** Expression of Langerin and CD103 was analyzed in population I (MHC II^high^ CD11c^+^) on day 2 p.i. **(B, C)** cervical lymph node cells were isolated from Langerin-DTR mice that were treated with diphtheria toxin (DT) or not as indicated (B) or from *Batf3*-/- and littermate controls (C) on day 7 p.i. and re-stimulated *in vitro* with DC^1940^ pulsed with heat killed (h.k.) *C*. *albicans* or left unpulsed for 5h in presence of Brefeldin A. IL-17A production by CD3^+^ CD4^+^ cells was analyzed by intracellular cytokine staining. Symbols represent individual mice, the mean + SD is indicated. All data are representative of at least 2 independent experiments.

To further dissect the heterogeneity of the MHC II^hi^ CD11c^+^ population in the cervical lymph nodes we performed staining with antibodies to Langerin and CD103. We detected two small populations consisting of Langerin^+^ CD103^+^ cells, most likely belonging to the Batf3-dependent DC subset and Langerin^+^ CD103^–^ Langerhans cells (Fig 5A). A third major subset consisted of Langerin^−^CD103^–^ cells. We therefore set out to determine the role of these different cell types in the induction of antifungal T cell response during OPC. We used Langerin-DTR mice to deplete Langerin-expressing cells. Administration of diphtheria toxin prior to infection with *C*. *albicans* did not alter the extent of *C*. *albicans*-induced Th17 priming in the cervical lymph nodes, which was assessed by measuring IL-17 production after re-stimulation of effector T cells with cognate antigen in vitro (Fig 5B). The *C*. *albicans*-induced Th17 cell response was also not affected in *Batf3*-deficient animals (Fig 5C), indicating that both CD103^+^ and CD103^–^ Langerin-expressing DCs were not essential for the induction of antifungal Th17 immunity in the oral mucosa.

**Fig 6:**
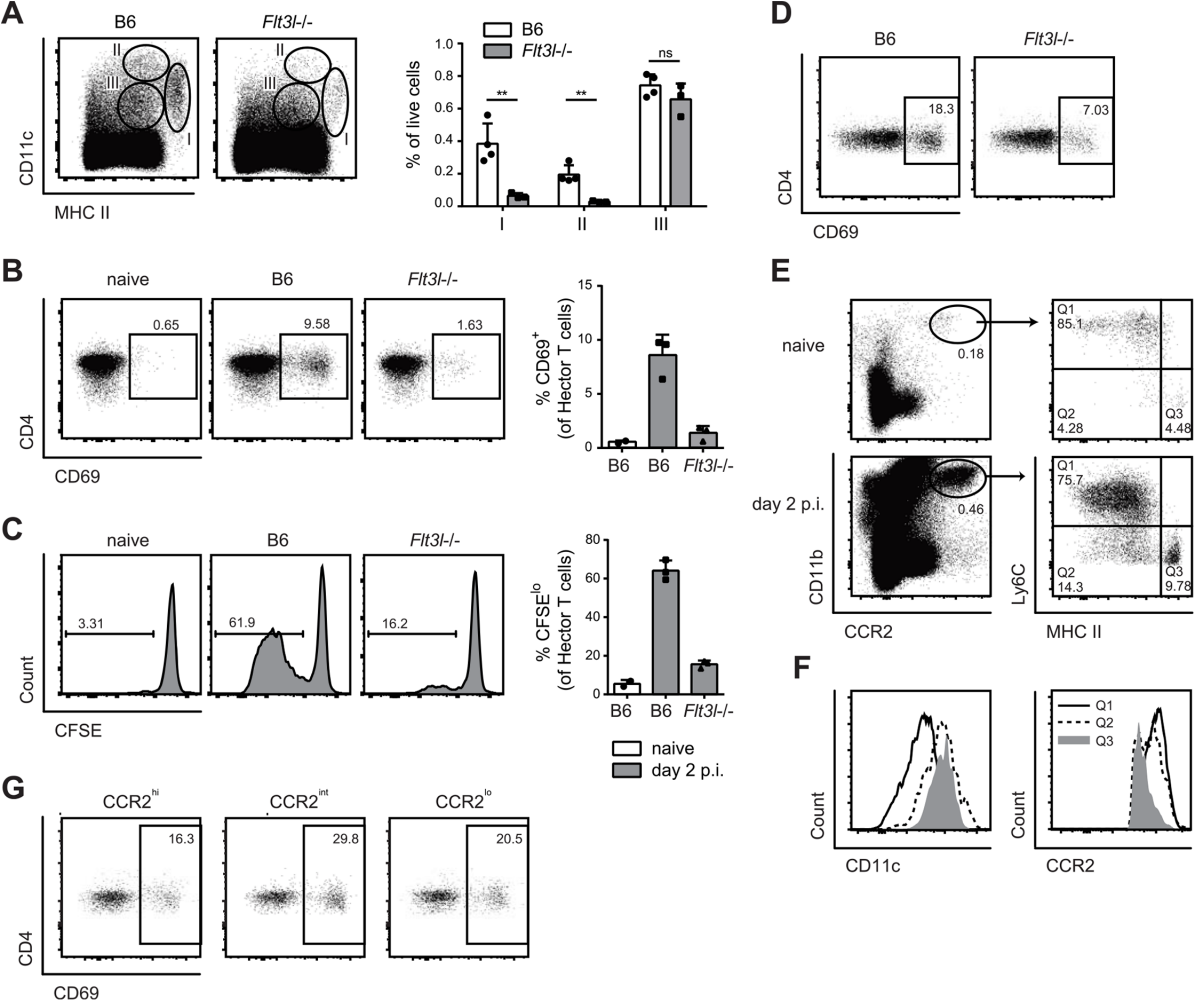
Flt3L-dependent migratory DCs and monocyte-derived DCs both present *C*. *albicans* derived antigen. **(A)** Cervical lymph node cells of B6 or *Flt3l*-/- mice were analyzed on day 2 post-infection. Representative FACS plots from individual mice and quantification of MHC II^high^ CD11c^+^ (population I), MHC II^+^ CD11c^high^ (population II) and MHC II^+^ CD11c^+^ cells (population III) from one of two independent experiments are shown. **(B, C)** Cervical lymph node cells were isolated from naïve B6 mice and from infected B6 and *Flt3l*-/- mice on day 2 post-infection, enriched for CD11b^+^ cells and co-cultured with CD4^+^ Hector T cells. Thy1.1^+^ CD3^+^ CD4^+^ TCRVα2^+^ cells were analyzed for CD69 expression after 1 day (B) and for proliferation after 4 days, respectively (C). Representative plots are shown on the left, summary of data from individual mice with mean + SD are shown on the right. Each symbol represents one mouse. Data are representative of 2 independent experiments. (**D**) As in B, but MHC II^high^ CD11c^+^ migratory DCs were isolated by FACS-sorting from the cervical lymph nodes of infected B6 and *Flt3l*-/- mice. (**E—F**) CCR2^+^ CD11b^+^ cells in the cervical lymph nodes of naïve and infected B6 mice on day 2 post-infection were analyzed for the expression of MHC II and CD11c (D). Three distinct subsets of CCR2^+^ CD11b^+^ cells (MHC II^lo/int^ Ly6C^+^ (Q1), MHC II^int^ Ly6C^-^ (Q2) and MHC II^high^ Ly6C^-^ (Q3)) from infected mice were further analyzed for the expression of CCR2 and CD11c (E). Representative FACS plots are shown. **(G)** Cervical lymph nodes were isolated from infected B6 mice on day 2 post-infection. CCR2^hi^, CCR2^int^ and CCR2^lo^ subsets within the MHC II^high^ CD11c^+^ population I were FACS-sorted and co-cultured with CD4^+^ Hector T cells for 1 day. Thy1.1^+^ CD3^+^ CD4^+^ TCRVα2^+^ cells were then analyzed for CD69 expression. Representative FACS plots are shown, Data are representative of 2 independent experiments.

The large Langerin^−^CD103^–^ subset of migratory DCs in the cervical lymph node appeared to be phenotypically homogeneous for all markers analyzed (Fig 4B). However, the subset may still comprise phylogenetically and functionally distinct cell types, including Flt3-dependent conventional DCs and monocyte-derived DCs that are Flt3-independent but dependent on Csf1R signaling for differentiation from inflammatory monocyte [13]. Consistent with this notion, we found that in *Flt3l*-/- mice, migratory MHC II^hi^ CD11c^+^ DCs, which include the large population of Langerin^−^CD103^–^ cells, were strongly reduced in *Flt3l*-/- mice compared to B6 mice (Fig 6A). Furthermore, the CD11b^+^ cervical lymph node cells isolated from infected *Flt3l*-/- mice were strongly impaired in their ability to induce CD69 upregulation and proliferation of Hector T cells *in vitro* (Fig 6B and 6C). This became also clear when the APC fraction was purified by FACS sorting: antigen presentation by Flt3L-dependent migratory DCs strongly promoted the activation of CD4^+^ Hector T cells *in vitro*, it was however not essential for the response (Fig 6D), indicating that Flt3L-independent migratory DCs are also involved. Thus, Flt3L-dependent migratory DCs appear to be an important source of antigen in cervical lymph nodes for T cell activation in response to *C*. *albicans* oral infection.

To assess the role of the Flt3L-independent monocyte-derived DCs we took advantage of the notion that these cells express CCR2 [12]. First, we noticed that CCR2^+^ monocytes, which are also Ly6C^hi^ and CD11b^hi^, rapidly accumulated in the infected tongue (S5 Fig). From day 2 post-infection, a proportion of CCR2^+^ monocytes up-regulated CD11c and MHC II and down-regulated Ly6C, indicating that they differentiated to DCs (S5 Fig). Second, CCR2^+^ monocytes were also found in the cervical lymph nodes of infected mice (Fig 6E), and some of them differentiated to DCs, as observed in the peripheral tissue (Fig 6E and 6F). We also observed that monocyte-derived DCs with the strongest expression of MHC II and CD11c had reduced levels of CCR2 (Fig 6F). Because of these characteristic markers, these cells fell in the gate of migratory MHC^hi^ CD11c^hi^ DCs (population I) and were hardly distinguishable from other migratory DCs such as Flt3L-dependent DCs.

To directly assess the antigen presentation capacity of the CCR2^+^-derived migratory DCs during OPC, we separated MHC II^hi^ CD11c^+^ DCs from the cervical lymph nodes of infected mice into three fractions according to their CCR2 expression and exposed them to CD4^+^ Hector T cells *in vitro*. We observed T cell activation with all the subsets, irrespective of their degree of CCR2 expression, suggesting that both monocyte-derived DCs (included mainly in the CCR2^hi^ and CCR2^int^ fractions) as well as CCR2^lo^ DCs, most likely reflecting Flt3L-dependent DCs, could directly present *C*. *albicans*-derived antigen (Fig 6G).

### Monocyte-derived and Flt3L-dependent migratory DCs complement each other for Th17 priming during OPC

**Fig 7:**
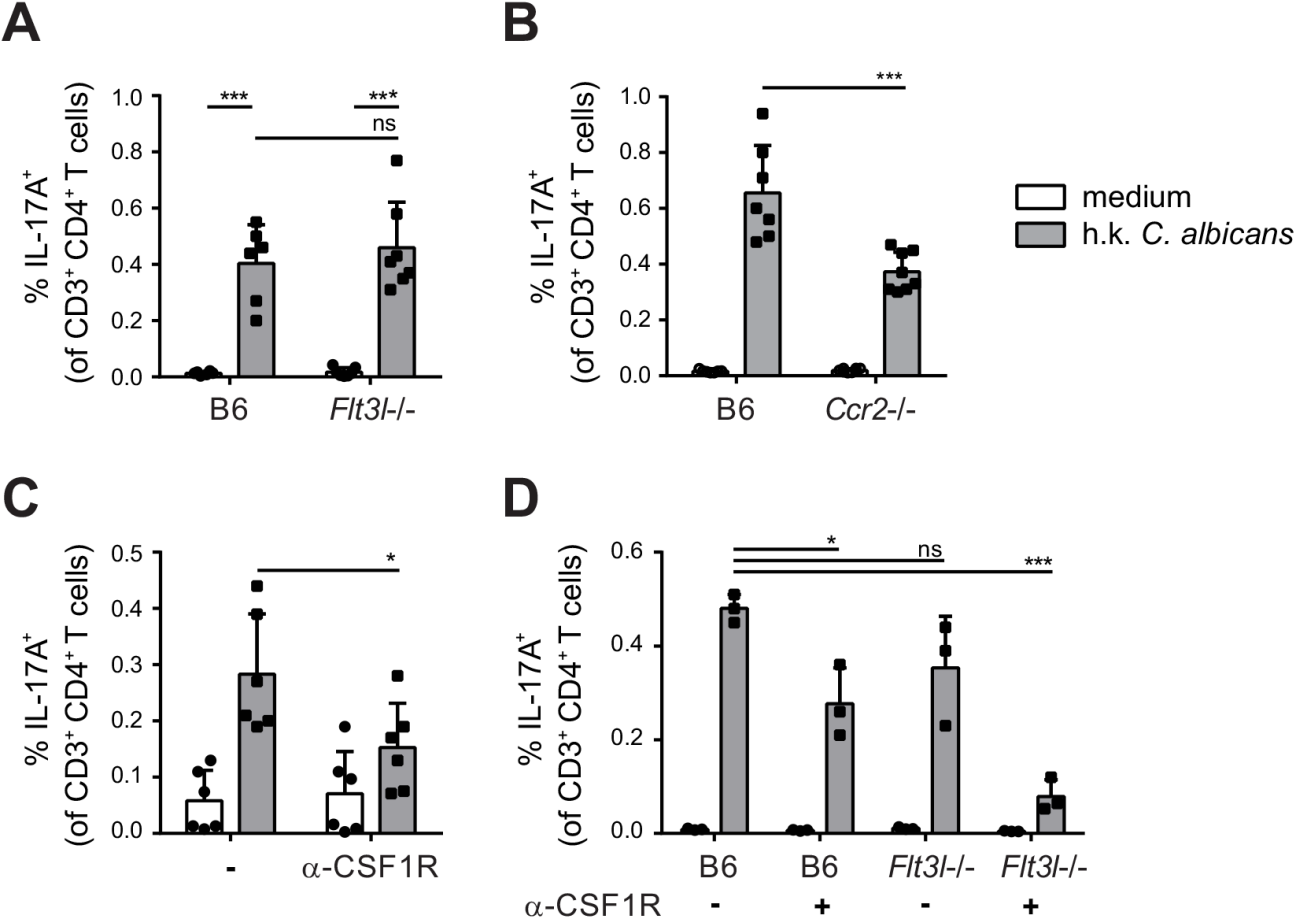
Flt3L-dependent migratory DCs and monocyte-derived DCs complement each other for Th17 priming during OPC. **(A)** Cervical lymph nodes were isolated from B6 and *Flt3l*-/- mice on day 7 post-infection and re-stimulated with heat-killed (h.k.) *C*. *albicans* or medium only. IL-17A production by endogenous CD3^+^ CD4^+^ cells was then analyzed by flow cytometry. (**B**) B6 and *Ccr2*-/- mice were infected sublingually and IL-17 production by endogenous CD3^+^ CD4^+^ cells was analyzed on day 7 post-infection as described in (A). (**C**) B6 mice were treated with anti-CSF1R or left untreated prior to sublingual infection with *C*. *albicans*. IL-17 production by endogenous CD3^+^ CD4^+^ cervical lymph node cells was analyzed on day 7 post-infection as described in (A). (**D**) B6 and *Flt3l*-/- mice were treated with anti-CSF1R or left untreated prior to sublingual infection with *C*. *albicans*. IL-17 production by endogenous CD3^+^ CD4^+^ cervical lymph node cells was analyzed on day 7 post-infection as described in (A). Each symbol represents one mouse, the mean + SD for each group is shown.

To evaluate the relative contribution of CCR2-dependent and Flt3L-dependent migratory DC subsets in T cell priming during OPC *in vivo*, we examined the induction of *C*. *albicans*-specific Th17 cell in the cervical lymph nodes of OPC infected *Flt3l*-/- and *Ccr2*-/- mice on day 7 post-infection. While the response was not affected by the absence of Flt3L-dependent DCs (Fig 7A), the frequency of IL-17-secreting *C*. *albicans*-specific CD4^+^ T was strongly reduced in *Ccr2*-/- mice compared to B6 controls (Fig 7B). Similarly to the endogenous response, adoptively transferred Hector T cells were also strongly impaired in their capacity to differentiate into IL-17-producing effector cells in *Ccr2*-/- mice (S6 Fig). Alterations in DC and monocyte populations may impair the innate control of the fungus; and an increased fungal burden can augment the extent of the T cell response. Therefore, to exclude the possibility that the results were influenced by differences in the available amount of antigen between the experimental groups, we treated the mice with Fluconazole from day 2 post-infection, a time point when the fungal burden was high and similar in all groups of mice (S7 Fig). This resulted in comparable weight recovery (S7 Fig) and clearance of the fungus to undetectable levels within the period of the experiment, which is indicative of comparable infection control in all experimental groups.

To further support the critical role of monocyte-derived DCs in Th17 priming during OPC, we made use of a CSF1R-specific antibody, which was shown to block the differentiation of monocytes into monocyte-derived DCs [13] and led to a significant reduction of CCR2^+^ cells within the migratory DC population (S8 Fig). Th17 induction in response to OPC was considerably lower in mice that were treated with this antibody prior to and during infection than in untreated controls (Fig 7C). However, similarly to what we observed in the *Ccr2*-/- mice, the reduction in Th17 response in mice treated with anti-CSF1R antibody was only about 50% compared to the response in controls. This could be explained by inefficient/incomplete blockade with the antibody, while in *Ccr2*-/- mice, monocyte trafficking may be partially compensated by CCR2-independent mechanisms. The impact of monocyte-derived DCs on Th17 priming during OPC may thus be underestimated in our system. An explanation for the partial effect could also be the involvement of other subsets of APCs that might even compensate to a certain extent for the absence of monocyte-derived DCs to promote Th17 priming in anti-CSF1R treated or CCR2-deficient mice. Because Flt3L-dependent DCs contribute to the presentation of *C*. *albicans*-derived antigen, at least when presentation was assessed in our *in vitro* assay (Fig 6B and 6C), we tested the hypothesis that they may play a relevant role in Th17 induction in absence of monocyte-derived DCs. For this we analyzed again the Th17 response to *C*. *albicans* in Flt3L-deficient animals and we included a group, in which we blocked the CSF1R prior to infection. All animals were again treated with Fluconazole from day 2 post-infection to minimalize differences in fungal burden between the experimental groups that could affect T cell priming (S7 Fig). This resulted in a nearly complete blockade of the *C*. *albicans*-specific Th17 response (Fig 7D). Together, our results demonstrate a contribution of different subsets of APCs to the Th17 response during OPC and highlight the critical role of monocyte-derived DCs and to a lesser extent Flt3L-dependent DCs in this process. While Flt3L-dependent DCs were not essential under normal conditions when monocyte-derived DCs were present, they did critically contribute if these primary APCs were absent.

## Discussion

Th17 cells have emerged as a key component of protective immunity against mucocutaneous candidiasis. In recent years, *Candida*-specific T cells have been studied in detail in humans and in mice leading to the identification of Th17-inducing innate pathways [9,10,36–40], revealing *Candida*-derived antigenic epitopes [31,41–43], and providing detailed insights into their clonal diversity [44]. Here we provide new insights in the mechanism that regulates fungus-specific T cell immunity. We generated a *Candida*-specific TCR transgenic mouse model to provide a tool for the detection, enumeration and characterization of antigen-specific T cells during infection *in vivo*. Using this new tool we identified the cellular players that present fungal antigen and instruct the Th17 response against *C*. *albicans* during oropharyngeal infection. CCR2-dependent monocytes-derived DCs rapidly accumulate in the oral mucosa during infection and directly present *Candida*-derived antigen in the draining lymph nodes. However, they are partially redundant for Th17 induction during OPC and complemented by tissue-resident Flt3-dependent migratory DCs for antifungal T cell priming *in vivo*. Together, this reveals a complex regulation of the antifungal T cell response by a multifaceted network MNPs in the oral mucosa.

T cells of the newly established Hector mouse model are responsive to a native epitope of *C*. *albicans* derived from the metabolic enzyme ADH1 that localizes to the fungal cell wall and is also abundant in biofilms [45]. ADH1 was reported previously to have immunogenic properties because it bears some structural homology to integrins and can mediate adherence to extracellular matrix [46], and ADH1-specific antibodies were detected in *Candida*-exposed individuals and mice [47,48]. Here we show that ADH1 determines T cell specificity ADH1 is highly conserved in diverse species of the genus *Candida* and in other Saccharomycotina. The short stretch of amino acids that define the T cell epitope identified here are 100% conserved in multiple clinically relevant species of *Candida* including *C*. *albicans*, *C*. *dubliniensis* and *C*. *glabrata*. The Hector mouse model thus offers new opportunities for investigating T cell responses against distinct species of *Candida*. Here we explored the regulation of *C*. *albicans*-specific T cells during oropharyngeal infection.

The oral cavity is the entry port and the first site of contact with the host for a multitude of microbes. Diverse DC subsets including tissue resident DCs as well as inflammatory DCs accumulating in the oral mucosa in response to infection and inflammation orchestrate T cell outcomes in response to these microbes. DC subsets in the oral mucosa are related to those described in other barrier tissues. However, their relative abundance and distribution as well as their putative function partially differs. Similarly to the skin, Langerhans cells are present in the oral mucosa, but in contrast to what was reported for an epicutaneous model of candidiasis, in which Langerhans cells were shown to be necessary and sufficient for antifungal Th17 priming [49], they are not essential for Th17 induction during OPC. Moreover, while Batf3-dependent DCs promote Th1 and inhibit Th17 cell differentiation during epicutaneous candidiasis [49], absence of Batf3-dependent cells does not affect the T cell response during OPC. Whether monocyte-derived DCs are involved in the T cell response against *C*. *albicans* in the skin had not been examined to our knowledge. The discrepancies observed between the two distinct tissues are likely explained by site-specific differences, including the important difference in abundance of Langerhans cells between the skin and the tongue, but may also be influenced by the different mouse models of Langerhans cell deficiency used in the two studies. In the oral mucosa, Langerhans cells seem to be mainly tolerogenic [17]. The observed differences in regulation of T cell priming at different sites underline the compartmentalization of the immune response against one and the same fungal organism in different tissue environments. This effect is even more pronounced when antifungal immunity in barrier tissues is compared with the response elicited during systemic candidiasis, which is dominated by type 1 immunity [50]. Whether the quality of the Th17 response primed in the oral mucosa versus the skin may differ, remains to be established. In the oral mucosa, *C*. *albicans*-specific T cells form a stable long-lived population of memory Th17 cells that efficiently responds to secondary infection [51]. Similarly, Th17 cells primed in the skin have recently been shown to enhance protection from epicutaneous fungal challenge with *C*. *albicans* [50].

The DCs presenting *C*. *albicans*-derived antigen to T cells in the cervical lymph nodes of OPC-infected mice belong to the CCR7-dependent migratory population. Distinguishing monocyte-derived DCs from other migratory DC subsets in the cervical lymph nodes of *C*. *albicans*-infected mice on the basis of their phenotype is difficult as they gradually lose their characteristic expression of Ly6C and CCR2. We therefore used an approach to separate them on the basis of their phylogenetic origin. Direct assessment of antigen presentation by migratory subsets revealed that both monocyte-derived DCs and Flt3L-dependent DCs presented *C*. *albicans*-derived antigen in the cervical lymph nodes of infected mice. They were however redundant for Th17 differentiation *in vivo*. Interrupting the differentiation of monocyte into DCs in absence of Flt3-dependent DCs almost abolished T cell priming, supporting the notion that other Flt3-independent DCs such as Langerhans cells are not required and not sufficient for Th17 induction during OPC.

Monocyte-derived DCs gain increasing attention for their role as professional APC to promote T cell responses [52–54] including those elicited by fungi such as *Aspergillus fumigatus* or *Blastomyces dermatitidis* [55,56]. In addition to priming adaptive immunity, CCR2-dependent cells also contribute to innate immunity against fungi including *C*. *albicans* and *A*. *fumigatus* [57,58]. The mechanism by which these cells contribute to acute protection has not been fully established. During OPC, innate lymphoid cells and innate lymphocytes provide important sources of IL-17 during the early phase of infection [59,60]. Whether monocytes and/or monocyte-derived DCs impact on the regulation of innate IL-17 secretion has not yet been established. Here we show that monocyte-dependent DCs, together with Flt3L-dependent DCs, orchestrate the antigen-specific T cell response to a clinically highly relevant fungal pathogen, and may thus have implications for potential future immunotherapeutic approaches and vaccine development against mucosal candidiasis.

## Supporting Information

S1 Fig Characterization of Hector TCR transgenic mice. (**A—B**) CD3^+^ CD4^+^ TCR β^+^ T cells from the spleen, cervical lymph nodes and thymus of naïve Hector and transgene-negative control mice (B6) were analyzed by flow cytometry for TCR Vα2 and Vβ4 expression. Representative FACS plots of splenic T cells are shown in A. Summary graphs with 6 individual mice are shown in B. (**C**) The proportion of CD4^+^ and CD8^+^ cells within the CD3^+^ TCRβ^+^ T cell population was analyzed in the spleen, cervical lymph nodes and thymus of naïve Hector and transgene-negative control mice (B6). For the thymus, the frequency of CD4^+^ CD8^+^ double positive (DP) T cells is also indicated. Each symbol represents an individual mouse, the mean ± SD is indicated. Data are pooled from two independent experiments. Note that the different ratio of CD4^+^: CD8^+^ T cells in spleen and lymph nodes of Hector mice is expected due to allelic exclusion of non-transgenic TCRβ genes and preferential lineage decision towards CD4^+^ T cells, as observed in other TCR transgenic mice [61,62].
(EPS)

S2 Fig Identification and characterization of the epitope recognized by Hector T cells in *Candida* spp. and *S*. *cerevisiae*. (**A**) Duplicate wells of hybridoma cells were stimulated with DC^1940^ cells that were pulsed with individual 15-mer peptides and IL-2 secretion by the hybridoma cells was quantified with the CTLL2 bioassay. The horizontal line indicates the detection limit of the assays (CTLL2 cells without IL-2 stimulation). (**B**) CFSE-labelled CD4^+^ Hector T cells were stimulated with splenocytes that were pulsed with decreasing concentrations of the C2, C3 and D1 peptide, respectively, and analyzed for proliferation by flow cytometry after 4 days of co-culture. (**C**) Hybridoma cells were cultured with DC^1940^ cells that were pulsed with decreasing amounts of heat-killed yeast cells and IL-2 secretion by the hybridoma cells was quantified by the CTLL2 bioassay. *C*. *a*., *C*. *albicans*; *C*. *d*., *C*. *dubliniensis*; *C*. *t*., *C*. *tropicalis*; *C*. *g*., *C*. *glabrata*; *C*. *k*., *C*. *krusei*; *S*. *c*., *S*. *cerevisiae*. Strain numbers refer to our internal strain collection. *C*. *albicans* strains SC5314 was included in all panels as a reference.
(EPS)

S3 Fig Th17 differentiation of *C*. *albicans*-specific Hector T cells during OPC. 10^5^ or 10^6^ CD4^+^ Hector T cells were adoptively transferred, as indicated, one day prior to sublingual infection with *C*. *albicans*. Cytokine production by Thy1.1^+^ CD4^+^ Vα2^+^ Hector cells on day 7 post-infection was analyzed by flow cytometry after re-stimulation with DC^1940^ cells pulsed with heat-killed (h.k.) *C*. *albicans* or pADH1_126-140_ as indicated. Percentage (A–B) and absolute numbers per mouse (C–D) of IL-17- (A, C) and IFN-γ-producing cells (B, D) are shown. Each symbol represents an individual mouse, the mean is indicated, data are pooled form 2 independent experiments.
(EPS)

S4 Fig IL-6 is expressed preferentially by the MHC II^hi^ CD11c^+^ migratory DC population. Cervical lymph node cells from OPC infected mice were analyzed for IL-6 expression by intracellular cytokine staining and FACS analysis. Populations I, II and III were identified as indicated in Fig 4. Numbers indicate the mean ± SD of IL-6^+^ cells within each population. n = 5.
(EPS)

S5 Fig Monocytes and monocyte-derived DCs accumulate rapidly in the oral mucosa. (**A**) Accumulation of Ly6G^+^ CCR2^-^ neutrophils and CCR2^+^ Ly6G^-^ monocytes in the tongue was analyzed at the indicated time points post-infection. Cells are pre-gated on CD11b^+^ cells. A representative plot from day 1 post-infection is shown on the left, and the summary from three individual mice of one representative experiment is shown on the right. **(B)** CCR2^+^ Ly6G^-^ cells in the tongue were analyzed for expression of MHC II and CD11c on indicated time points post-infection. A representative plot from day 1 post-infection is shown on the left, and quantification of MHC II^+^ CD11c^-^, MHC II^+^ CD11c^+^ and MHC II^-^ CD11c^-^ subpopulations are shown on the right. Data are mean + SD of 3 independent mice and representative of 2 independent experiments.
(EPS)

S6 Fig The response of Hector T cells is strongly impaired in *Ccr2*-/- mice. (**A—B**) CD4^+^ Hector T cells were adoptively transferred into *Ccr2*-/- and B6 control mice one day prior to sublingually infection with *C*. *albicans*. Cervical lymph node cells were isolated on day 7 post-infection, re-stimulated with DC^1940^ cells pulsed with heat-killed (h.k.) *C*. *albicans* or pADH1_126-140_ peptide or left unpulsed and IL-17A and IFN-γ production by CD3+ CD4+ Thy1.1+ TCRVα2+ Hector T cells was analyzed by flow cytometry. Representative FACS plots are shown in (A), the summary of data from individual mice with mean + SD is shown in (B).
(EPS)

S7 Fig OPC-infected mice display high fungal burden on day 2 and normal weight recovery within 5 to 7 days post-infection. (**A**) Fungal burden in the tongue of OPC-infected B6, *Ccr2*-/- and *Flt3l*-/- mice as well as B6 and *Flt3l*-/- mice treated with anti-CSF1R antibody on day 2 post-infection. The dotted line indicates the detection limit. (**B—E**) Weight curves from B6 and *Flt3l*-/- mice (B), B6 and *Ccr2*-/- mice (C), B6 mice treated or not with anti-CSF1R (D), and B6 and *Flt3l*-/- mice treated or not with anti-CSF1R (E) that were included in the experiments shown in Fig 7A–7D.
(EPS)

S8 Fig B6 mice were treated with anti-CSF1R or left untreated prior to sublingual infection with *C*. *albicans*, as described in Fig 7C. The CCR2^+^ subset within the MHC-II^hi^ migratory DC population was analyzed on day 2 post-infection.
(EPS)
